# Facile Design of Low-Dimensional, Hybrid Transparent Conductors Achieving Efficient, Scalable All-Solution-Processed Sb-Chalcogenide-Based Semitransparent Solar Cells

**DOI:** 10.1021/acsami.6c01899

**Published:** 2026-06-09

**Authors:** Thanh Tai Nguyen, Atanas Katerski, Arvo Mere, Malle Krunks, Nicolae Spalatu, Ilona Oja Acik

**Affiliations:** Laboratory for Thin Film Energy Materials, Department of Materials and Environmental Technology, 54561 Tallinn University of Technology, Ehitajate tee 5, Tallinn 19086, Estonia

**Keywords:** antimony sulfide, transparent photovoltaics, ultrasonic spray pyrolysis, AgNWs, solution-process

## Abstract

Antimony sulfide (Sb_2_S_3_), an emerging photovoltaic material, is desirable for efficient, cost-effective solar cells, attributed to its capability to achieve highly crystallized Sb_2_S_3_ films by nonvacuum techniques, favoring the design of all-solution-processed photovoltaics. A high-performance Sb_2_S_3_ solar cell often constitutes hydrophobic hole-transporting layers, creating a surface energy mismatch with hydrophilic solution-processed, effective transparent conductors (TCs) like silver nanowires (AgNWs). Therefore, the realization of an efficient, all-solution-processed Sb_2_S_3_ solar cell remains challenging. Herein, a completely solution-processed Sb_2_S_3_ solar cell is achieved by designing an effective AgNW-based TC by intermixing an AgNW solution with 27 vol % poly­(3-hexylthiophene) (P3HT). An interaction between isopropyl alcohol, a polar solvent in the AgNW solution, and P3HT results in the aggregation of the polymer, enhancing adhesion between the AgNWs and the glass/FTO/TiO_2_/Sb_2_S_3_/P3HT surface. This technique enables a reduction in the sheet resistance of AgNW-based TCs by 88%. The Sb_2_S_3_-based solar cells with modified AgNW-based TCs provide an efficiency of 2.1% and a consistent open-circuit value of 0.7 V for up-scaled devices. The adaptation of TCs allows the device to work in bifacial mode with a see-through feature, evidenced by an average visible light transmittance of 11.7%. The developed all-solution-processed Sb_2_S_3_-based semitransparent photovoltaics would be advantageous for photovoltaic-integrated product applications.

## Graphical abstract

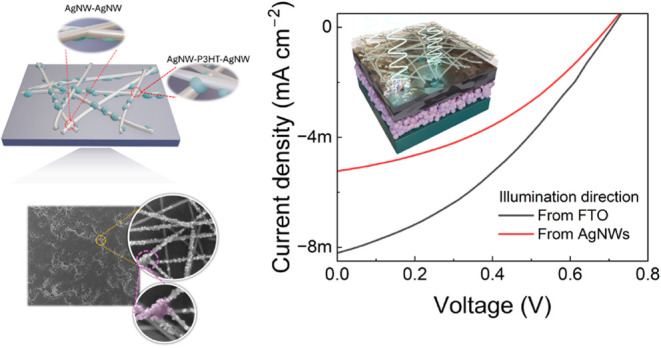

## 1 Introduction

Photovoltaics is the leading technology to achieve sustainable renewable energy, aiming to replace fossil fuels, which have a negative impact on the environment and limited resources.^1^ The transition of energy consumption from fossil fuels to renewable energy has been triggered through the adoption of solar cells in power generation, with the ultimate target of photovoltaics-integrated grids.^2,3^ To reach this target, significant research effort has been spent on reducing the cost per watt of solar cells through two approaches, including increasing device efficiency and adopting low-cost photovoltaic materials.^4^ Thin-film solar cells with reasonable efficiency for low material consumption are desirable for the latter approach. Various thin-film materials have been developed for effective photovoltaic applications, such as CdTe, CIGS, perovskite, and Sb_2_S_3_.^5–8^ Among these materials, Sb_2_S_3_ is particularly promising for advancing thin-film photovoltaics due to its high absorption coefficient (>10^4^ cm^–1^), anisotropic conducting properties, chemical stability, and eco-friendliness^9–11^ The research on Sb_2_S_3_-based solar cells has witnessed rapid progress recently, with an increase in the number of publications and improvements in device performance.^12^

High-quality Sb_2_S_3_ thin films, achieved by vacuum and solution-processed techniques, are essential for high-performance thin-film solar cells. Vacuum methods such as magnetron sputtering and rapid thermal evaporation are usually characterized by sophisticated maintenance and high power consumption, attributed to the involvement of high vacuum and temperature processes.^13,14^ Meanwhile, solution-processed methods are known for the simple setup and low energy consumption, resulting from non/low-vacuum operation.^5,15^ Recently, the record performance of Sb_2_S_3_-based solar cells (power conversion efficiency (PCE) of 8.3%) has been achieved with the Sb_2_S_3_ layer fabricated by the solution-processed technique.^16^ Therefore, Sb_2_S_3_ by solution processing can further reduce the cost of solar cells while providing high device performance. Various solution-processed methods, such as chemical bath deposition, hydrothermal techniques, spray pyrolysis, and spin-coating, have been proposed to completely explore the potential of Sb_2_S_3_ for thin-film photovoltaics.^17–20^ Ultrasonic spray pyrolysis (USP) is an emerging solution-processed technique that can enable a high yield of large-scale and uniform Sb_2_S_3_ thin films.^21,22^ Importantly, this technique allows robust control of Sb_2_S_3_ properties with high effectiveness in raw material consumption. Specifically, USP can realize solar cells with an efficiency of 7.5% for an air-stable, single-phase Sb_2_S_3_.^23^ This value is comparable to the high-performance Sb_2_S_3_-based solar cells fabricated by chemical bath deposition (PCE of 8%), which is the most commonly used technique for synthesizing Sb_2_S_3_ thin films.^5,9^ Thus, USP-based Sb_2_S_3_ holds promising potential for developing high-performance thin-film solar cells.

In addition to the advantages of the synthesizing method, the wide bandgap property (1.7–1.8 eV) can facilitate Sb_2_S_3_ for semitransparent and tandem solar cell applications.^9^ Developing Sb_2_S_3_-based semitransparent photovoltaics (STPVs) will provide new perspectives on utilizing photovoltaic technology for power generation. STPVs with the capability of transmitting visible light can be integrated as structural parts of buildings, electronic devices, and vehicles. This approach will further trigger the transition in energy consumption with the focus on urbanized areas where the land budget for solar cell farms is limited.^24^ To realize STPVs, designing effective transparent electrodes is essential. Gold (Au) as a back contact is mostly used in designing highly efficient Sb_2_S_3_-based solar cells.^12,23,25^ Sb_2_S_3_-based STPVs have been realized by adopting a thin Au layer with a thickness of 10 nm.^26^ However, the relatively high cost along with thermal evaporation, a vacuum-based fabrication, would hinder the Au-based transparent electrode from developing cost-effective, scalable Sb_2_S_3_-based solar cells. In addition to designing transparent electrodes, researchers are attempting to develop a wide bandgap hole transporting layer to enhance the device’s transparency; however, few studies report on fully transparent Sb_2_S_3_-based solar cells.^10,25,27^ Therefore, more research effort is required to realize effective Sb_2_S_3_-based STPVs.

Silver nanowire (AgNW)-based transparent conductors (TCs) are adopted in the present study for the first time to design all-solution-processed Sb_2_S_3_-based STPVs to complement the target of cost-effective, scalable thin-film solar cells. AgNW TCs possess the highest electrical conductivity compared to other solution-processed TCs, such as polymers and carbon nanotubes.^28,29^ AgNWs are commonly dispersed in hydrophilic solvents, such as isopropyl alcohol (IPA), for stable dispersion and uniform distribution of AgNWs.^30,31^ Spin-coating an IPA-based AgNW solution on a hydrophilic substrate, such as poly­(3-hexylthiophene) (P3HT), can induce a surface energy difference between the AgNWs and the P3HT layer.^32,33^ This mismatch would provide an uneven distribution of the AgNW network and poor electrical connection between AgNWs and P3HT, resulting in poor photogenerated power extraction of Sb_2_S_3_-based STPVs with P3HT as a hole transporting layer. The current study presents an approach for improving the uniformity and electrical properties of the AgNW network over the hydrophobic substrate by intermixing the AgNW solution with P3HT. The added P3HT functions as an adhesion promoter, enabling better AgNW distribution on the photovoltaic structure along with enhanced connectivity among individual AgNWs. This effect results in a reduction in sheet resistance in the AgNW network by 88%. The P3HT-AgNW (PAgNW)-based TCs exhibit a high visible light transmittance of 73.2% (at a wavelength of 550 nm). The integration of PAgNWs with Sb_2_S_3_-based photovoltaics results in all-solution-processed STPVs with a see-through feature and a PCE of 2.1%. Additionally, the study provides a prototype of large-scale glass/FTO/TiO_2_/Sb_2_S_3_/P3HT/PAgNW STPVs for electrically powered window applications, suggesting the potential of USP for developing cost-effective, scalable thin-film solar cells.

## 2 Experimental Section

### 2.1 Materials

The following materials were used: glass/FTO substrate (7 Ω sq^–1^); titanium­(IV) tetraisopropoxide (TTIP, 99 wt %, Acros Organics); acetylacetone (99 wt %, Acros Organics); ethanol (96.6 vol %, Estonian Spirit); methanol (99.9 vol %, Sigma-Aldrich); antimony trichloride (99.99 wt %, Sigma-Aldrich); thiourea (99 wt %, Sigma-Aldrich); chlorobenzene (99.5 vol %, Sigma-Aldrich); poly­(3-hexylthiophene 2,5-diyl) (P3HT) 100 kDa, > 90% regioregular (Sigma-Aldrich); and AgNWs in isopropyl alcohol (IPA) (5 mg mL^–1^, Sigma-Aldrich).

### 2.2 Solar Cell Fabrication

Sb_2_S_3_ solar cells were fabricated in a configuration (glass/FTO/TiO_2_/Sb_2_S_3_/P3HT/AgNWs). The fabrication process started with cleaning the glass/FTO substrate with DI water and IPA in an ultrasonic bath for 10 min each. TiO_2_ and Sb_2_S_3_ were deposited by the USP system. TiO_2_ was sprayed from a solution mixture of 0.2 M TTIP with 0.2 M acetylacetone in ethanol for 30 min on a hot plate maintained at a temperature of 360 °C. The sprayed TiO_2_ samples were then annealed at 450 °C for 30 min in air. Sb_2_S_3_ thin films were spray-deposited from a solution mixture of 60 mM SbCl_3_ and 180 mM thiourea in methanol. The films were deposited on glass/FTO/TiO_2_ placed on a hot plate temperature of 198 °C.^20^ The as-deposited Sb_2_S_3_ films were annealed at 320 °C for 6 min under nitrogen. The P3HT layer was formed by spin-coating P3HT solution (11 mg P3HT in 1 mL chlorobenzene) on glass/FTO/TiO_2_/Sb_2_S_3_. The deposited P3HT was activated by annealing at 170 °C for 5 min under nitrogen. The AgNW layer was formed by spin-coating P3HT–AgNW mixtures with various added P3HT volume percentages. The mixture was dried at ambient conditions. A glass/FTO/TiO_2_/Sb_2_S_3_/P3HT/Au device was fabricated by thermal evaporation of Au.

### 2.3 Characterization

The crystal structure of the annealed Sb_2_S_3_ thin films was examined using a Rigaku Ultima IV X-ray diffractometer with a Cu Kα source (λ = 1.5406 Å). The optical absorption and transmittance measurements were made using a Jasco V-670 ultraviolet–visible spectrophotometer (UV–Vis) in the range of 250–1000 nm. A Zeiss HR FESEM was used to analyze the cross-sectional and surface morphologies of the layers. The current–voltage characteristics of the glass/FTO/TiO_2_/Sb_2_S_3_/P3HT/AgNW devices were measured using AUTOLAB PGSTAT 30 coupled with a Wavelabs LS-2 LED solar simulator with an AM1.5G (100 mW cm^–2^) light source. A Newport 69911 system with a 300 W xenon lamp was used to measure the external quantum efficiency (EQE) of the fabricated solar cells. The sheet resistance of AgNW-based transparent conductors was measured at room temperature using MMR’s variable-temperature Hall system and van der Pauw controller H-50. The impedance spectroscopy of Sb_2_S_3_-based solar cells was measured using an AUTOLAB PGSTAT 30 equipped with a frequency analyzer module. The measurement was conducted for a frequency range of 0.1 MHz to 10 Hz, a scanned potential of −0.3 to 0.3 V, and an applied 10 mV of AC perturbation.

## 3 Results and Discussion

Figure 1a presents a schematic of all solution-processed fabrication of Sb_2_S_3_-based STPVs. A glass/FTO with a high visible light transmittance (>75%) (Figure S1, Supporting Information) was adopted as a substrate to fabricate Sb_2_S_3_-based STPVs. An electron-transporting layer, TiO_2_, and an ultrathin absorber, Sb_2_S_3_, were synthesized by the USP method. The successful deposition of TiO_2_ and Sb_2_S_3_ layers was confirmed by a distinguished difference in the layer distribution obtained by a cross-sectional SEM image, as shown in Figure 1b. The thickness of the Sb_2_S_3_ layer on glass/FTO/TiO_2_ was less than 100 nm, which was controlled by the number of cycles for spraying the USP system. The controlled thickness was desirable for developing STPVs.^34^ However, it was challenging to grow a continuous Sb_2_S_3_ layer with a thickness below 100 nm on TiO_2_, as evidenced by the uncovered TiO_2_ by the Sb_2_S_3_ layer, shown in Figure 1c. A strong bonding energy of Ti–O (662 kJ mol^–1^) and the inert properties of TiO_2_ favor the growth of the Sb_2_S_3_ layer according to the Volmer–Weber mechanism, inducing an island-like morphology.^35,36^ Despite the discontinuity, the USP method enabled a large grain size of Sb_2_S_3_ film with a relative scale of 1.5 μm. A large grain size is necessary for effective free-carrier movement within the Sb_2_S_3_ layer, which is required for high-performance Sb_2_S_3_ STPVs. The obtained morphological characteristics were consistent with a previous report on the growth of Sb_2_S_3_ on TiO_2_ by USP.^22,23^ The quality of the Sb_2_S_3_ layer was further assessed by measuring the XRD profile of the glass/FTO/TiO_2_/Sb_2_S_3_ sample. The XRD pattern, presented in Figure 1d, revealed the crystallized structure of the TiO_2_ and Sb_2_S_3_ layers on the FTO substrate, exhibiting the inherent characteristics of the heat-treated TiO_2_ and Sb_2_S_3_ layers grown by the USP technique.^20^ Importantly, the XRD profile of the Sb_2_S_3_ layer possessed (hk1) planes ((211) and (221)), which are essential for fast charge transport across the Sb_2_S_3_ layer.^9^

**Figure 1:**
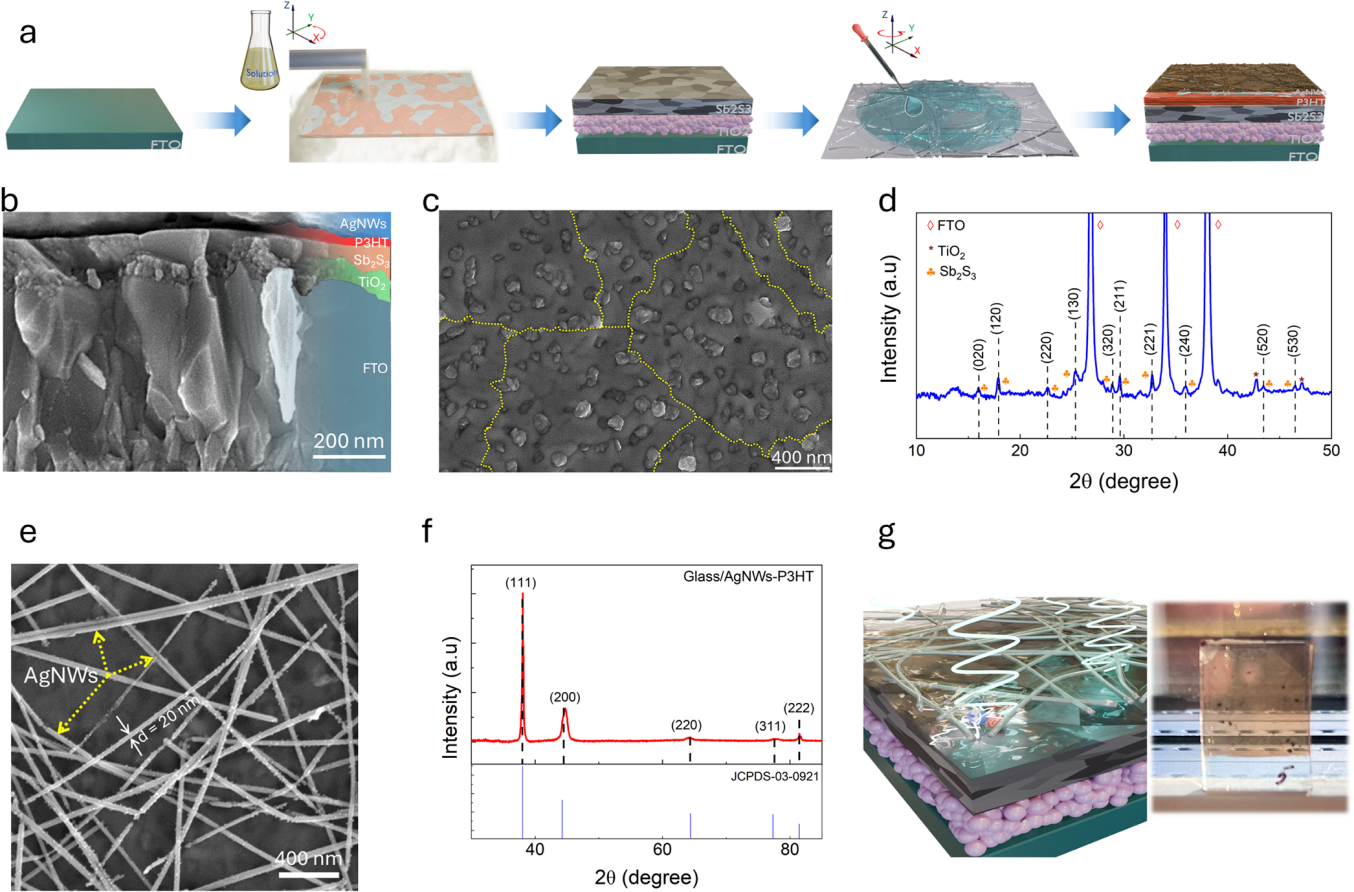
(a) Schematics of all-solution-processed Sb_2_S_3_-based semitransparent solar cells. (b) Cross-section SEM image of the glass/FTO/TiO_2_/Sb_2_S_3_/P3HT/AgNW device. (c) Top-view SEM image of glass/FTO/TiO_2_/Sb_2_S_3_. (d) XRD profiles of the glass/FTO/TiO_2_/Sb_2_S_3_structure. (e) Top-view SEM image of glass/FTO/TiO_2_/Sb_2_S_3_/P3HT/AgNWs. (f) XRD profile of glass/PAgNWs. (g) Schematics of the glass/FTO/TiO_2_/Sb_2_S_3_/P3HT/AgNW device for front illumination, accompanied by the digital image of device.

A hole-transporting layer, P3HT, and a transparent conductor, AgNWs, were sequentially deposited on glass/FTO/TiO_2_/Sb_2_S_3_ by the spin-coating method to complete the STPV design with a structure of glass/FTO/TiO_2_/Sb_2_S_3_/P3HT/AgNWs. FTO and AgNWs served as the front and back electrodes, respectively. The spin-coating method is preferred for solution-processed TC regarding its simplicity and ability to provide high film uniformity.^29,37^ Figure 1e presents a top-view SEM image of Sb_2_S_3_-based STPVs, revealing the nanomesh structure of the AgNW-based TC with an individual AgNW diameter of 20 nm. The characterization also found a conformal coating of the hole transporting layer over the glass/FTO/TiO_2_/Sb_2_S_3_ surface. The conformity of the P3HT layer prevented direct contact between AgNWs and TiO_2_, which can induce a shunting problem in Sb_2_S_3_-based STPVs. Furthermore, the uniformity of P3HT over the 100 nm Sb_2_S_3_ layer would favor the electrical properties of Sb_2_S_3_ solar cells to follow a p-i-n structure, which is advantageous for achieving a high built-in potential and a large depletion region width.^5^ The XRD analysis revealed a cubic structure of the AgNWs, exhibiting a similar dominant peak intensity indexed to the (111) plane with a reference profile (JCPDS 03-0921), as shown in Figure 1f. The nanomesh configuration will allow an illuminating light to penetrate the AgNW network and interact with the photovoltaic structure, TiO_2_/Sb_2_S_3_/P3HT, resulting in photogeneration processes in the STPVs, as shown in Figure 1g. This characteristic is desirable for deploying Sb_2_S_3_ solar cells in bifacial glass/FTO substrate (7 Ω sq–1), titanium­(IV) tetraisopropoxide (TTIP)–99 wt % (Acros Organics), acetylacetone–99 wt % (Acros Organics), ethanol–96.6vol % (Estonian Spirit), methanol–99.9 vol % (Sigma-Aldrich), antimony trichloride–99.99 wt % (Sigma-Aldrich), thiourea–99 wt % (Sigma-Aldrich), chlorobenzene–99.5 vol % (Sigma-Aldrich), poly­(3-hexyl-thiophene-2,5-diyl) (P3HT) 100 kDa, > 90% regioregular (Sigma-Aldrich), and AgNWs in isopropyl alcohol (IPA) (5 mg mL^–1^, Sigma-Aldrich). operation to improve solar cell performance. Furthermore, the adaptation of AgNWs as TCs allows the Sb_2_S_3_ solar cell to be semitransparent (digital image of the device, Figure 1g), which is essential for photovoltaic-integrated buildings and tandem solar cell applications.

The effect of the added P3HT on the optoelectronic properties of the AgNW-based TC was initially assessed by measuring the transmittance spectra of the glass/PAgNWs with various added P3HT concentrations, as shown in Figure 2a. The addition of P3HT concentrations of 0, 13.5, 27, and 41 vol % into the IPA-based AgNW solution was labeled as PAgNW-0, PAgNW-1, PAgNW-2, and PAgNW-3, respectively. In addition to the glass/PAgNWs, a transmittance profile of glass/P3HT was also presented. The PAgNWs exhibited broad light transmittance over a spectrum ranging from 400 to 850 nm. Additionally, the transmittance profiles of PAgNWs showed a distinguished difference with glass/P3HT by a transmittance maximum located in the ultraviolet light region. The observed transmittance maxima were attributed to a plasmon resonance effect of AgNWs in the ultraviolet spectrum.^38^ The visible light transmittance (at a wavelength of 550 nm) of the TC dropped from 81.3% to 67.7% with an increase of P3HT concentration in the AgNW solution from 0% to 41 vol %. A noticeable transmittance hump was observed for a wavelength range of 610–700 nm for P3HT concentrations larger than 13.5 vol %, representing an inherent transmittance feature of P3HT. These optical characteristics indicated a successful integration of P3HT and AgNWs.

**Figure 2:**
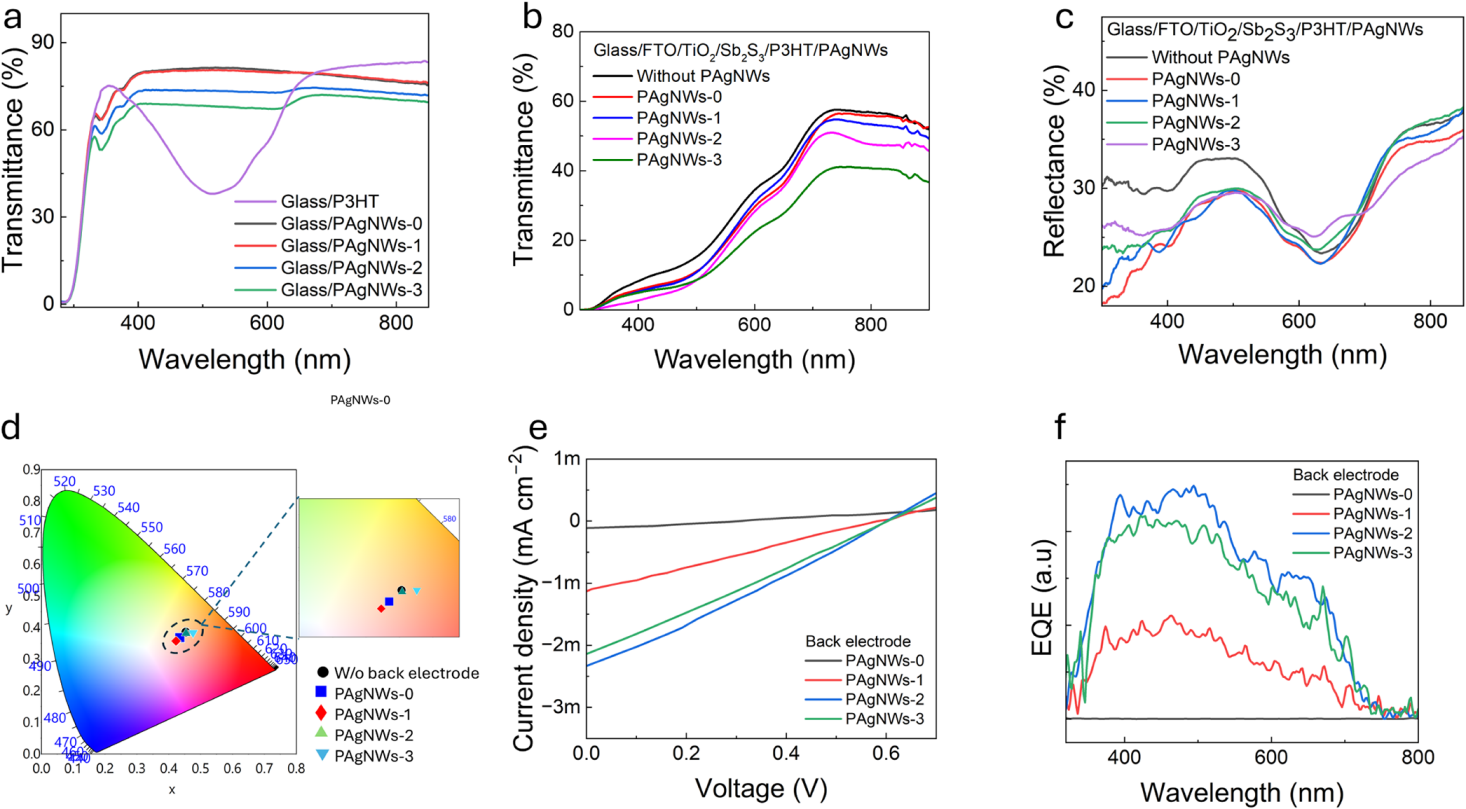
(a) Transmittance profiles of glass/PAgNWs and glass/P3HT. (b) Transmittance profile of glass/FTO/TiO_2_/Sb_2_S_3_/P3HT/PAgNWs for different PAgNW-based TC designs. (c) Reflectance profiles of Sb_2_S_3_-based STPVs for different PAgNW-based TC designs . (d) CIE plot of the coordinates of Sb_2_S_3_-based STPVs. (e) *I–V* characteristics of Sb_2_S_3_-based STPVs under 1 Sun illumination. (f) External quantum efficiency (EQE) profiles of Sb_2_S_3_-based STPVs.

Figure 2b presents the transmittance profile of glass/FTO/TiO_2_/Sb_2_S_3_/P3HT/PAgNWs for various designs of PAgNW-based TCs. An average visible transmittance (AVT) was estimated by the following relation:^39^

1 $$AVT=\frac{\int_{380nm}^{825nm}T\left(\lambda \right)P\left(\lambda \right)S\left(\lambda \right)d\lambda }{\int_{380nm}^{825nm}P\left(\lambda \right)S\left(\lambda \right)d\lambda }$$

where *T*(λ), *P*(λ), and *S*(λ) are the transmittance spectra of the STPVs, solar spectra (AM 1.5G), and the photonic response of the human eye, respectively. The glass/FTO/TiO_2_/Sb_2_S_3_/P3HT exhibited an AVT of 26.5%. The value slightly dropped to 22.7%, 21.9%, 20.2%, and 16.7% with PAgNW-based TCs for P3HT concentrations of 0, 13.5, 27, and 41 vol %, respectively. The reduction in device transparency indicated a successful integration of AgNWs with the glass/FTO/TiO_2_/Sb_2_S_3_/P3HT structure. Digital images of the Sb_2_S_3_-based STPVs (Figure S2, Supporting Information) showed enhanced uniformity of the PAgNW layer over glass/FTO/TiO_2_/Sb_2_S_3_/P3HT with the increase of added P3HT concentration. The slight decrease in device transparency for PAgNW-based TCs with P3HT concentrations less than 41 vol % suggested that the visible light transmittance of Sb_2_S_3_-based STPVs was governed by the glass/FTO/TiO_2_/Sb_2_S_3_/P3HT structure. The AgNW network is commonly characterized by an inhomogeneous distribution of AgNWs with open spaces in the network. The open spaces allow the light to be transmitted through the AgNW network, allowing the slight drop in AVT values of the Sb_2_S_3_-based STPVs. In addition to optical transparency, reflectance is an essential parameter for exploring Sb_2_S_3_-based STPVs for bifacial operation. The reflectance of the STPVs was suppressed by 9% with the deposited PAgNW-based TCs for the light spectra of 300–620 nm, as presented in Figure 2c, which was desirable for bifacial illumination applications. The nanostructure of PAgNW-TCs can induce light scattering, resulting in the suppression of reflectance associated with Sb_2_S_3_-based STPVs. The adaptation of PAgNWs as TCs allowed a color analysis of Sb_2_S_3_-based STPVs according to the International Commission on Illumination (CIE), as presented in Figure 2d. Accompanying transparency, color is an important aesthetic parameter in designing effective integrated photovoltaic products.^40^ The color of Sb_2_S_3_-based STPVs was evaluated by adopting the CIE 1931 standard. The standard implements XYZ tristimulus values for describing color,

2 $$X=\frac{\int_{360nm}^{830nm}T\left(\lambda \right)P\left(\lambda \right)x\left(\lambda \right)d\lambda }{\int_{360nm}^{830nm}P\left(\lambda \right)y\left(\lambda \right)d\lambda }$$

3 $$Y=\frac{\int_{360nm}^{830nm}T\left(\lambda \right)P\left(\lambda \right)y\left(\lambda \right)d\lambda }{\int_{360nm}^{830nm}P\left(\lambda \right)y\left(\lambda \right)d\lambda }$$

4 $$Z=\frac{\int_{360nm}^{830nm}T\left(\lambda \right)P\left(\lambda \right)z\left(\lambda \right)d\lambda }{\int_{360nm}^{830nm}P\left(\lambda \right)y\left(\lambda \right)d\lambda }$$

where *x*(λ), *y*(λ), and *z*(λ) are red, green, and blue color-matching functions, respectively. The x and y coordinates of the CIE diagram were estimated according to the following relations:

5 $$x=\frac{X}{X+Y+Z}$$

6 $$y=\frac{Y}{X+Y+Z}$$

The chromaticity coordinates of Sb_2_S_3_-based STPVs with PAgNW-based TCs for P3HT concentrations of 0, 13.5, 27, and 41 vol % were (0.434, 0.368), (0.423, 0.358), (0.454, 0.383), and (0.476, 0.386), respectively. The estimated coordinates indicated the color deviation of the STPV device from the standard white spectrum—D65 (0.325, 0.315), suggesting the suitability of the device for color photovoltaic applications. Interestingly, the device with PAgNW-2 preserved the color characteristics of the FTO/TiO_2_/Sb_2_S_3_/P3HT sample.

The effectiveness of the developed PAgNW-based TCs as a back contact was initially characterized by measuring current–voltage (*I–V*) characteristics of the Sb_2_S_3_-based STPVs under 1 Sun illumination conditions, as shown in Figure 2e. Increasing the P3HT concentration in PAgNWs resulted in enhanced performance of the STPVs, especially the short-circuit current (*J*_*SC*_). The *J*_*SC*_ value enhanced from 111.6 μA cm^–2^ to 2.3 mA cm^–2^ when the P3HT concentration increased from 0 to 27 vol%. However, *J*_*SC*_ reduced with a further increase in P3HT concentration. It is worth mentioning that 1 coat of PAgNWs was applied for the characterization. While a noticeable modification in *J*_*SC*_ was obtained, there was a negligible difference in the open-circuit voltage (*V*_OC_) value when the P3HT concentration increased from 13.5 to 41 vol %. The Sb_2_S_3_-based STPVs with PAgNWs exhibited a *V*_OC_ value of around 0.61 V. The influence of PAgNWs on *J*_*SC*_ of Sb_2_S_3_-based STPVs was further assessed by measuring the external quantum efficiency (EQE) of the device, as presented in Figure 2f. The obtained EQE profiles exhibited consistency with the measured *J*_*SC*_ values of the Sb_2_S_3_-based STPVs. A weak EQE response was received for the device with PAgNW-0. The EQE response was enhanced with higher P3HT concentrations in the PAgNWs and achieved the strongest response for the device with PAgNW-2. The EQE profiles exhibited a cutoff wavelength of around 730 nm, attributed to the Sb_2_S_3_ bandgap of 1.7 eV. It is important to note that the light illumination was on the FTO side of the device for the *I–V* and EQE characterizations.

The photon-active structure, glass/FTO/TiO_2_/Sb_2_S_3_/P3HT, was maintained with different designs of PAgNW back contact. Therefore, the increase in *J*_*SC*_ of Sb_2_S_3_-based STPVs, presented in Figure 2e, was governed by the electrical conductivity of PAgNW-based TCs. The electrical properties of PAgNWs were characterized by measuring the dark *I–V* characteristics of glass/P3HT/PAgNWs, as shown in Figure 3a. The *I–V* characteristics exhibited linear behavior following Ohm’s law for various P3HT concentrations, indicating that the added P3HT, a semiconductor, did not alter the ohmic properties of the AgNW network. The highest current value was obtained for PAgNW-2, with a value of 16.7 mA at a potential of 2 V. The highest current value of glass/P3HT/PAgNW-2 was attributed to the lowest sheet resistance value, as shown in Figure 3b. Sheet resistance profiles were obtained from the Hall measurement technique. The sheet resistance dropped from 595 Ω sq^–1^ for PAgNWs-0 to 72 Ω sq^–1^ for PAgNWs-2, indicating a reduction of sheet resistance by 88%. The reduction in the sheet resistance suggested an enhancement of the conductivity of PAgNWs with the increase of P3HT concentration. However, the further increase in the P3HT concentration of PAgNWs (over 27 vol %) resulted in a higher sheet resistance value.

**Figure 3:**
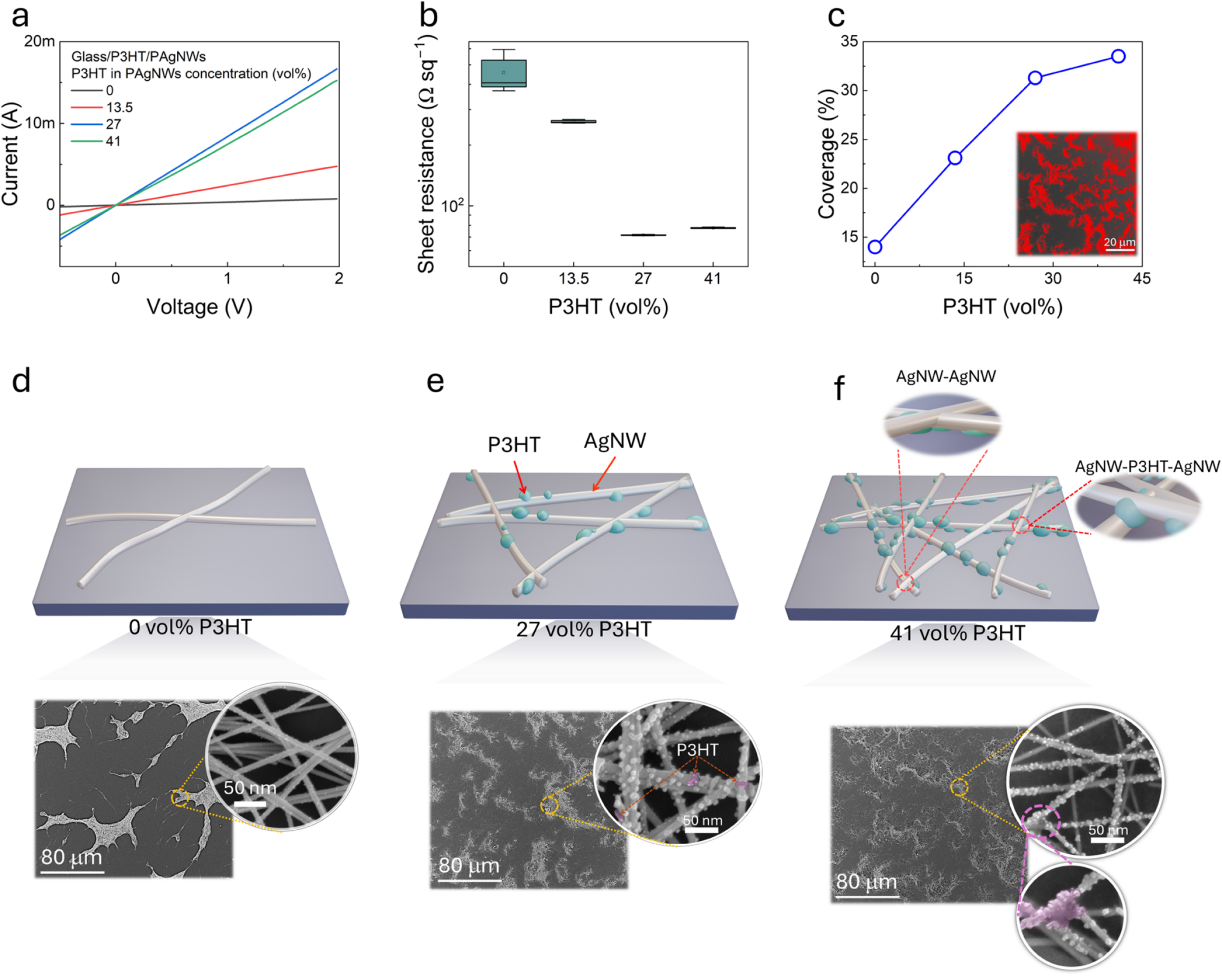
(a) *I–V* characteristics of glass/P3HT/PAgNWs. (b) Sheet resistance profiles of glass/P3HT/PAgNWs. (c) Coverage ratio of PAgNWs over the surface of glass/P3HT. Proposed conduction mechanism for glass/P3HT/PAgNWs with various added P3HT concentrations in AgNW solution: (d) 0 vol %, (e) 27 vol %, and (f) 41 vol %.

The conductivity of an AgNW network is not only a function of individual AgNW dimensions but also characterized by AgNW–AgNW contact properties.^28^ The AgNW network contains insulating macrovoids that limit its lateral conductivity. Therefore, carrier transport between individual AgNWs facilitates free carrier conduction through the AgNW network. An increase in the number of connections, reflected by an improvement in the coverage ratio of AgNWs over a surface, will enable better carrier charge transport. Figure 3c presents the coverage ratio of PAgNWs as a function of the added P3HT concentration. The coverage ratio, estimated by Jimage software, increased with the higher P3HT concentration and achieved the highest value of 33.5% for PAgNW-3. The addition of P3HT to the AgNW solution resulted in a higher conductivity of the AgNW network. Furthermore, improved coverage can facilitate more efficient photogenerated carrier collection of AgNWs from the glass/FTO/TiO_2_/Sb_2_S_3_/P3HT structures. The systematic increase of the coverage allowed for an understanding of the optical behavior of the Sb_2_S_3_-based STPVs around the optical band edge (1.7 eV). The higher coverage blocked the transmittance of light through the AgNW layer, resulting in a decrease in the transmittance of the devices, specifically for the wavelengths longer than 730 nm. Figure 3d–f proposes and presents a conduction mechanism to provide a deeper understanding of the charge transport behavior in the PAgNW network.

The PAgNWs without P3HT exhibited a low number of AgNW–AgNW connections, attributed to a poor AgNW distribution on glass/P3HT, which was evidenced by a top-view SEM image, as shown in Figure 3d. The hydrophilic property of an IPA-based AgNW solution can introduce a surface energy mismatch with a hydrophobic substrate like P3HT, resulting in poor coverage of the AgNW network over the P3HT surface. The low coverage ratio induced high sheet resistance, hindering the charge transport in the AgNW network. An addition of P3HT dissolved in chlorobenzene in AgNW solution created P3HT aggregations distributed on AgNWs, as presented by the magnified SEM image of the AgNW network associated with Figure 3e. It has been previously reported that polar solvents such as IPA can interact with P3HT via dipole–dipole interactions, promoting polymer aggregation.^41,42^ The aggregated P3HT acted as an adhesion promoter of AgNWs on the P3HT surface, resulting in a higher coverage ratio of the PAgNW network. The function of the adhesion promoter associated with P3HT was reflected by the tilted SEM image of glass/FTO/TiO_2_/Sb_2_S_3_/P3HT/PAgNWs (Figure S3, Supporting Information). Enhanced coverage enabled higher numbers of AgNW–AgNW contacts, favoring charge transport through the AgNW network, resulting in a reduction in sheet resistance of the TCs. A higher added P3HT concentration (41 vol %) resulted in better coverage of AgNWs over the P3HT surface; however, it increased the possibility of forming an AgNW-P3HT-AgNW connection. The AgNW-P3HT-AgNW connection was evidenced by a magnified SEM image, as presented in Figure 3f. The high resistivity of P3HT increased the AgNW–AgNW junction resistance, hindering free carrier transport through the AgNW network, resulting in an increase of sheet resistance associated with PAgNW-3.

In addition to electrical conductivity, it is important to investigate the influence of PAgNWs by spin-coating on the electrical properties of Sb_2_S_3_-based solar cells. The electrical characteristics of Sb_2_S_3_-based solar cells were assessed by the impedance spectroscopy technique. High-performance Sb_2_S_3_-based solar cells are often achieved with opaque Au electrodes obtained by the sputtering technique. In the context of impedance characterization, a solar cell with Au as a back contact was characterized along with PAgNW-based STPVs to identify the current limitations of Sb_2_S_3_-based STPVs. Au-based Sb_2_S_3_ solar cells had a PCE of 3.9% (Figure S4, Supporting Information). Because of the high sheet resistance and highly uneven distribution of PAgNW-0, Sb_2_S_3_-based STPVs with PAgNW-1 and PAgNW-2 were adopted for characterization to reveal the influence of added P3HT concentration on the device properties. Figure 4a presents the impedance profiles of the Sb_2_S_3_ solar cell in a Nyquist plot. The impedance of the device was smallest for the Au electrode. An increase in P3HT concentration resulted in reduced impedance for the device with the PAgNW electrode. A Bode plot of impedance profiles revealed one minimum and two minima for the device with Au and PAgNW electrodes, respectively, as shown in Figure 4b. A minimum in a Bode plot of phase indicates a charge transfer process, representing a resistance–capacitance constant in a photoelectric device.^43^ A Voigt circuit was applied to fit the impedance profiles of the Sb_2_S_3_ solar cell. The circuit included series resistance (R_S_), junction resistance (R_J_), and a constant phase element (Q). The constant phase element was used to consider inhomogeneity in the solar cell, such as material roughness and surface states.^44,45^ The impedance of the constant phase element was estimated by the following relation:

7 $$Z_{Q}=\frac{1}{Q^{T}{\left(i\omega \right)}^{Q^{P}}}$$

**Figure 4:**
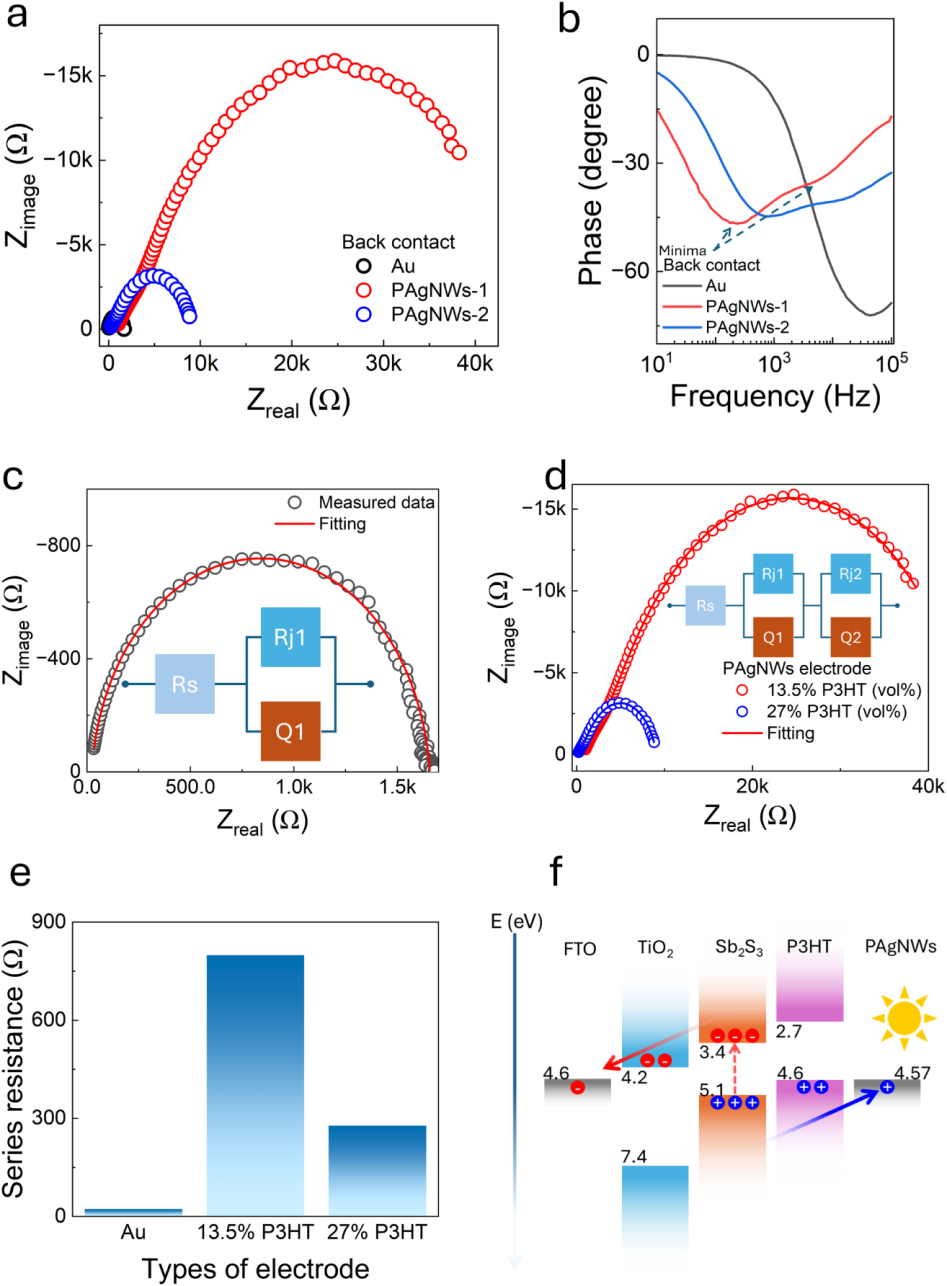
Impedance profiles of Sb_2_S_3_-based solar cells with various back contact designs: (a) Nyquist plot and (b) Bode plot. Fitting impedance profiles of (c) glass/FTO/TiO_2_/Sb_2_S_3_/P3HT/Au and (d) glass/FTO/TiO_2_/Sb_2_S_3_/P3HT/PAgNWs. (e) Series resistance of Sb_2_S_3_-based solar cells with various back contact designs. (f) Energy band diagram of FTO/TiO_2_/Sb_2_S_3_/P3HT/PAgNWs.

where i and ω are imaginary unit and cyclic frequency, respectively. Q^T^ and Q^P^ are two values of the constant phase element. Q^P^ value ranges from 0 to 1.

The equivalent circuit provides a satisfactory fit to the measured impedance data, as shown in Figure 4c and d. Table 1 presents the fitted parameters of the impedance spectra. The fitting revealed that the lowest R_S_ value of 23 Ω belonged to the Sb_2_S_3_ solar cell with the Au electrode, as shown in Figure 4e. R_S_ of the Sb_2_S_3_ solar cell included bulk resistance, interface resistance, and electrode resistance.^46^ Increasing the P3HT concentration in PAgNWs reduced R_S_; however, the value was still much higher compared to the device with the Au electrode. It is worth noting that recombination resistance, represented by R_J1_, was not suppressed by PAgNWs by the spin-coating method. Thus, it is important to further reduce R_S_ of the PAgNW-based STPVs by increasing the conductivity of the AgNW network to improve the performance of STPVs. The conductivity of AgNWs can be improved by reducing AgNW–AgNW contact resistance via overcoating the network with dielectric layers.^28^ The second R_J_//Q combination, as presented in Figure 4d, might present the P3HT (hole transporting layer)/PAgNW interface. The fitting information was contradictory to the energy band diagram, presented in Figure 4f, which indicated Ohmic characteristics between the P3HT layer and PAgNWs. The raised contradiction can result from the inhomogeneity in the electrical conductivity of the AgNWs, attributed to insulating macrovoids in the networks.^47,48^ The inhomogeneity was reflected by the low value of Q^P^, as shown in Table 1. Designing AgNW-based hybrid TCs can mitigate the contradiction. In this context, low-dimensional conduction, such as ultrathin metallic TCs, can combine with AgNWs to enhance the network’s lateral conductivity by turning the macrovoids from insulating to conducting.^49^

**Table 1:** Fitting Parameters of the Impedance Profiles of Sb_2_S_3_-Based Solar Cells for Various Back Contact Designs

Back contact	R_S_ (Ω)	Rj_1_ (kΩ)	Q_1_^T^ (nF)	Q_1_^P^	Rj_2_ (kΩ)	Q_2_^T^ (μF)	Q_2_^P^
Au	23	1.63	36	0.95			
PAgNWs-1	799.26	40.21	268	0.83	3.88	1.1	0.59
PAgNWs-2	120.56	7.82	409	0.84	1.14	2.19	0.59

Because of the enhanced optoelectrical properties, PAgNWs-2 was utilized to explore the capability of the USP method for developing scalable, bifacial Sb_2_S_3_-based STPVs. Figure 5a presents *I–V* characteristics of the Sb_2_S_3_-based STPVs for various numbers of coats of PAgNWs-2. Increasing the number of coats resulted in the enhanced performance of the device; however, the photovoltaic performance got saturated with 3 coats. The transparency of the device reduced with thicker PAgNWs-2 (Figure S5, Supporting Information); therefore, Sb_2_S_3_-based STPVs with two coats of PAgNWs-2 were used for the later discussions. The Sb_2_S_3_-based STPVs exhibited *J*_*SC*_ of 8.2 mA cm^–2^, *V*_OC_ of 0.71 V, and a fill factor (FF) of 36.5%, resulting in a PCE of 2.1%. The reproducibility of the device’s performance was investigated through statistical analysis, providing an average PCE of 2.1% (Figure S6, Supporting Information). The bifacial operation of the Sb_2_S_3_-based STPVs was confirmed by recording photogenerated *I–V* characteristics from front (glass/FTO side) and back (PAgNWs-2 side) illumination, as shown in Figure 5b. The device possessed a PCE of 1.4% for the front illumination condition. The bifacial photonic response of the device was further confirmed by strong EQE signals for front and back illuminations, as shown in Figure 5c. The drop in the EQE value for the back illumination, which was responsible for the reduction of *J*_*SC*_, was attributed to the relatively low visible light transmittance of PAgNWs-2-based TCs and parasitic absorption loss associated with the P3HT (hole transporting layer).^50^ The drop in *J*_*SC*_ of Sb_2_S_3_-based STPVs for the front illumination can be reduced by adopting a wide bandgap hole transporting layer^20^ and/or designing AgNW-based multilayer TCs for higher visible light transmittance.^49^

**Figure 5:**
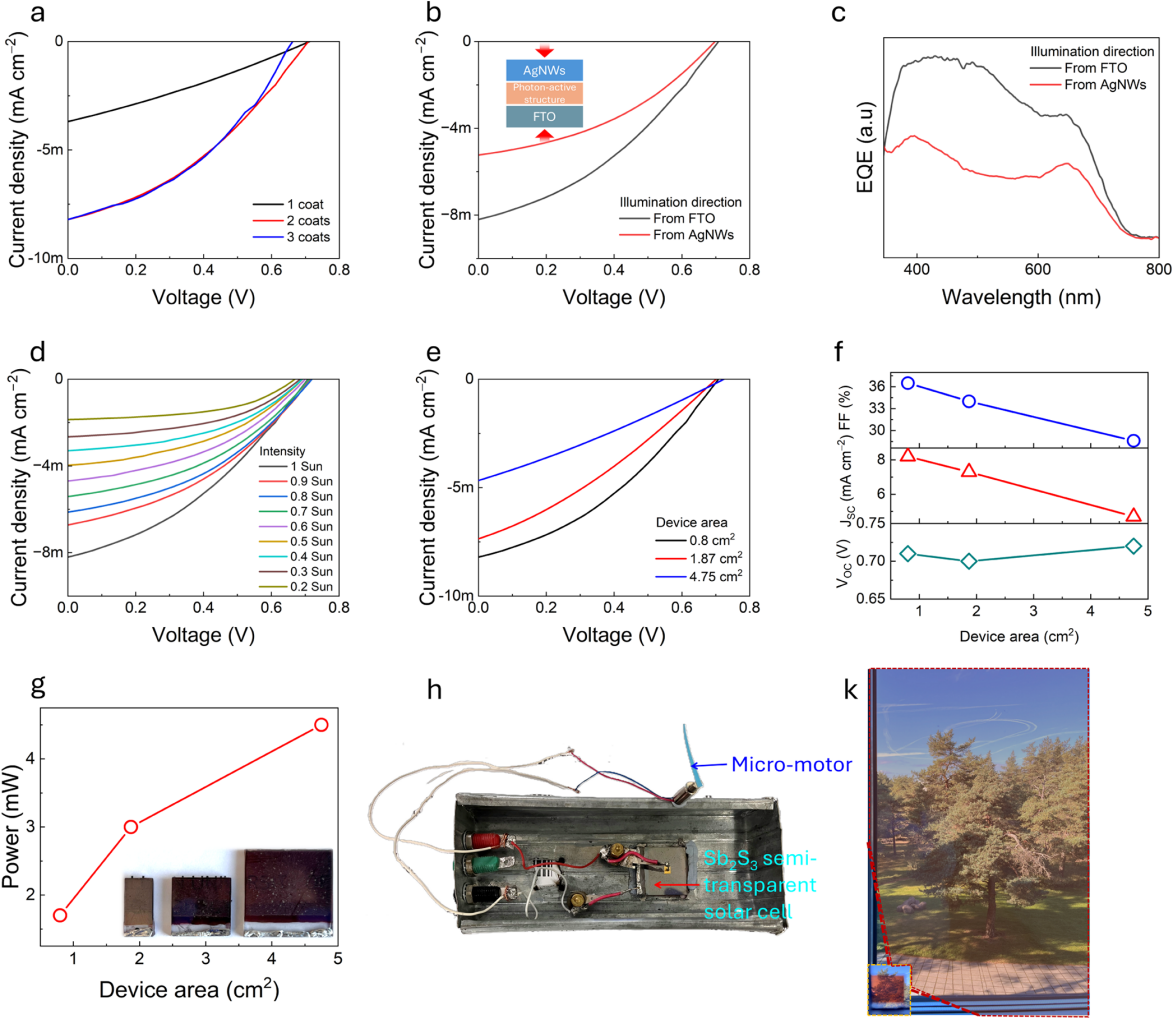
(a) *I–V* characteristics of glass/FTO/TiO_2_/Sb_2_S_3_/P3HT/PAgNW-2 under simulated 1 Sun illumination. (b) Bifacial photogenerated *I–V* characteristics of the Sb_2_S_3_-based STPVs. (c) Bifacial EQE spectra of the Sb_2_S_3_-based STPVs. (d) *I–V* characteristics of the Sb_2_S_3_-based STPVs as functions of light intensity. (e) Influence of the STPV area on the photogenerated *I–V* characteristics. (f) Photovoltaic parameters of Sb_2_S_3_-based STPVs as functions of the device area. (g) Photogenerated power of the Sb_2_S_3_-based STPVs as functions of the device area. (h) Experimental setup for demonstrating power generation of the Sb_2_S_3_-based STPVs. (k) Prospects of deploying the Sb_2_S_3_-based STPVs for photovoltaics-integrated buildings.

The photovoltaic behavior of Sb_2_S_3_-based STPVs was further characterized by measuring the *I–V* characteristics for different light illumination intensities. The device exhibited a stable photovoltaic response for a wide range of light intensities, ranging from 0.2 to 1 Sun, as shown in Figure 5d. Specifically, the device possessed photovoltaic parameters of 1.86 mA cm^–2^, 0.67 V, and an FF of 50.7%, representing a PCE of 3.2% for an illumination intensity of 0.2 Sun. The high photovoltaic sensitivity to low illumination intensity would suggest the suitability of the developed Sb_2_S_3_-based STPVs for emerging applications such as indoor photovoltaics.^51^ Additionally, *J*_*SC*_ was proportional to light intensity (P) according to the relation *J*_SC_ *∼ P* ^α^ (Figure S7, Supporting Information). The α value of 0.9 was close to unity, indicating that a less defective photovoltaic material was achieved by USP techniques.^52^ Leveraging the scale of solar cells is a critical requirement for the perspective of photovoltaics-integrated grids.^53^ The capability of the USP technique for scalable solar cells was explored through examining the photovoltaic performance of the Sb_2_S_3_-based STPVs with different photoactive areas, including 0.8, 1.87, and 4.75 cm^2^. The *I–V* characteristics of the devices under simulated 1 Sun illumination are presented in Figure 5e. Importantly, *V*_OC_ was consistent for various device areas, with a value around 0.7 V, as shown in Figure 5f, indicating the suitability of the USP technique for leveraging the scale of STPVs. *J*_*SC*_ and FF values showed a decreasing trend with larger device scales, possibly attributed to the reduced lateral conductivity of the PAgNWs over a large-scale deposition. The characterization implied that the performance of the leveraged-scale Sb_2_S_3_-based STPVs was governed by the conductivity of the PAgNW-based TCs.

Scalable Sb_2_S_3_-based STPVs by the USP method enabled higher photogenerated power, as presented in Figure 5g. Increasing the device area from 0.8 to 4.75 cm^2^ resulted in enhanced power generation from 1.7 to 4.5 mW. The photogenerated power was utilized to operate a micromotor with an experimental setup shown in Figure 5h to demonstrate a practical application of scalable Sb_2_S_3_-based STPVs. The successful operation of the micromotor (Video S1, Supporting Information) provides a perspective to integrate the developed Sb_2_S_3_-based STPVs as a photovoltaic window with buildings, as presented in Figure 5k. The progress of Sb_2_S_3_-based STPVs is presented in Table 2. It is important to note that most of the Sb_2_S_3_-based STPVs were achieved with Au as a back contact fabricated by sputtering technique, which is a vacuum-based technique. Jianwang reported a solution-processed STPV that has the structure ITO/CdS/Sb_2_S_3_/Spiro-OMeTAD/PEDOT:PSS/graphene.^54^ The utilization of CdS as an electron-transporting layer would raise a concern of toxicity. Additionally, graphene is not ideal for upscaling solar cells because the method to transfer it is complicated.^55^ Therefore, the proposed all-solution-processed Sb_2_S_3_-based STPVs in the present study would be advantageous for developing scalable, cost-effective, and sustainable STPVs. To properly evaluate the performance of solution-processed Sb_2_S_3_-based STPVs, the light utilization efficiency (LUE) of the device was estimated by the following relation:^24,56^

8 LUE=AVT×PVE

**Table 2:** Summarization of Sb_2_S_3_-Based Semi-Transparent Solar Cell Research Progress^a^

	Deposition method							
Structure	Sb_2_S_3_	Back contact	J_SC_ (mA cm^–2^)	V_OC_ (mV)	FF (%)	PCE (%)	AVT (%)	Ref.
ITO/TiO_2_/Sb_2_S_3_/P3HT/PEDOT:PSS/Au	ALD	TE	12.1	679	42.0	3.4	8	^26^
ITO/CdS/Sb_2_S_3_/Spiro-OMeTAD/PEDOT:PSS/graphene	HT	L	10.0	509	42.7	2.2	14	^54^
FTO/CdS/Sb_2_S_3_/CuSCN/Au	HT	SP	8.8	580	41.7	2.1	13.7	^25^
FTO/CdS/Sb_2_S_3_/ P3HT/PEDOT:PSS/Au	HT	SP	10.3	665	59.7	4.2	10.2	^34^
FTO/TiO_2_–ZnS/Sb_2_S_3_/ P3HT/PEDOT:PSS/Au	HT	SP	10.3	613	51.4	3.6	8.8	^34^
FTO/TiO_2_/Sb_2_S_3_/ P3HT/PEDOT:PSS/Au	SP	SP	10.3	558	54.2	3.1	10	^58^
FTO/TiO_2_/Sb_2_S_3_/CuSCN/ITO	SP	SP	8.1	511	48	2.5	11.8	^58^
FTO/TiO_2_/Sb_2_S_3_/ P3HT/PAgNWs	USP	S	8.2	710	36.5	2.1	11.7	This work

a Abbreviations: ALD, atomic layer deposition; TE, thermal evaporation; HT, hydrothermal; L, lamination; SP, sputtering; USP, ultrasonic spray; and S, spin-coating.

The LUE value of the Sb_2_S_3_ device was 24.6. The modest LUE value indicated room for improvement in solution-processed Sb_2_S_3_-based STPVs. The enhanced LUE value can be achieved by designing TCs composed of multilayers.^58^ A deployment of solution-processable wide bandgap dielectric materials such as ZnO, TiO_2_, and Al-doped ZnO as a layer above PAgNWs will maintain all solution-processed characteristics of the STPVs. The sheet resistance of PAgNWs can be reduced further by the PAgNWs/dielectric structure via tightening the AgNW connection by a capillary force mechanism, enabling lower Rs of the Sb_2_S_3_-based STPVs.^28^ The reduction in Rs will favor an enhancement of *J*_*SC*_ and FF, allowing higher PCE. Furthermore, the utilization of high refractive index dielectrics such as TiO_2_ (refractive index of 2.6 at a wavelength of 587 nm) would allow optical engineering of the Sb_2_S_3_-based STPVs by tuning the thickness of dielectric materials, opening the possibility for higher AVT values.^57,58^

Enhancing AgNWs’ conductivity through multilayer AgNW-based TC design can overcome the current limitations of *J*_*SC*_ and FF in the PAgNW-based STPVs.

## 4 Conclusion

A complete solution-processed STPV was achieved by enhancing the compatibility of AgNWs with the glass/FTO/TiO_2_/Sb_2_S_3_/P3HT structure. Enhanced compatibility was achieved by introducing P3HT into the hydrophilic AgNW solution. An interaction between IPA and P3HT by a dipole–dipole mechanism resulted in the aggregation of the P3HT polymer, which functioned as an adhesion promoter and enabled a better distribution of AgNWs over the surface of the hole transport layer. An enhanced coverage of AgNWs over the P3HT as a hole-transporting layer surface resulted in a significant reduction of the sheet resistance of AgNW networks by 88%. This effect enabled effective photogenerated power extraction from Sb_2_S_3_-based STPVs, evidenced by a PCE of 2.1%. Moreover, the scalable Sb_2_S_3_-based STPVs maintained a high *V*_OC_ value of 0.7 V for an Sb_2_S_3_ thickness of less than 100 nm. The developed all-solution-processed Sb_2_S_3_-based STPVs possessing an AVT value of 11.7% can deliver a photogenerated power of 4.5 mW, which operated a micromotor. The practical demonstration enabled a perspective on deploying Sb_2_S_3_-based STPVs for photovoltaic-integrated products such as buildings, electronic devices, and vehicles.

## Supplementary Material





## Notes

A listing of the contents of additional transmittance, digital images of devices, cross-sectional SEM images, *I*–*V* characteristics, and *J*_SC_ with light intensity dependence (PDF)
Successful operation of a micromotor (Video S1) (MOV)
