# Supplementary material for: Facile Design of Low-Dimensional, Hybrid Transparent Conductors Achieving Efficient, Scalable All-Solution-Processed Sb-Chalcogenide-Based Semitransparent Solar Cells
Source: ACS Appl Mater Interfaces. 2026 Jun 9;18(24):33981–92. doi: 10.1021/acsami.6c01899 (PMC13307073; doi:10.1021/acsami.6c01899)
Supplement: Supplementary file 1 [file am6c01899_si_001.pdf]

Supporting Information

Facile design of low dimensional, hybrid transparent conductor achieving efficient, scalable all-solution processed Sb-chalcogenide-based semi-transparent solar cells

Thanh Tai Nguyen[a], Atanas Katerski[a], Arvo Mere[a], Malle Krunks[a], Nicolae Spalatu[a], Ilona Oja Acik[a]**

[a]Laboratory for Thin Film Energy Materials, Department of Materials and Environmental Technology, Tallinn University of Technology, Ehitajate tee 5, Tallinn, 19086, Estonia

Corresponding author: Nicolae Spalatu (Email: nicolae.spalatu@taltech.ee*), : Ilona Oja Acik (Email:* *ilona.oja@taltech.ee*)

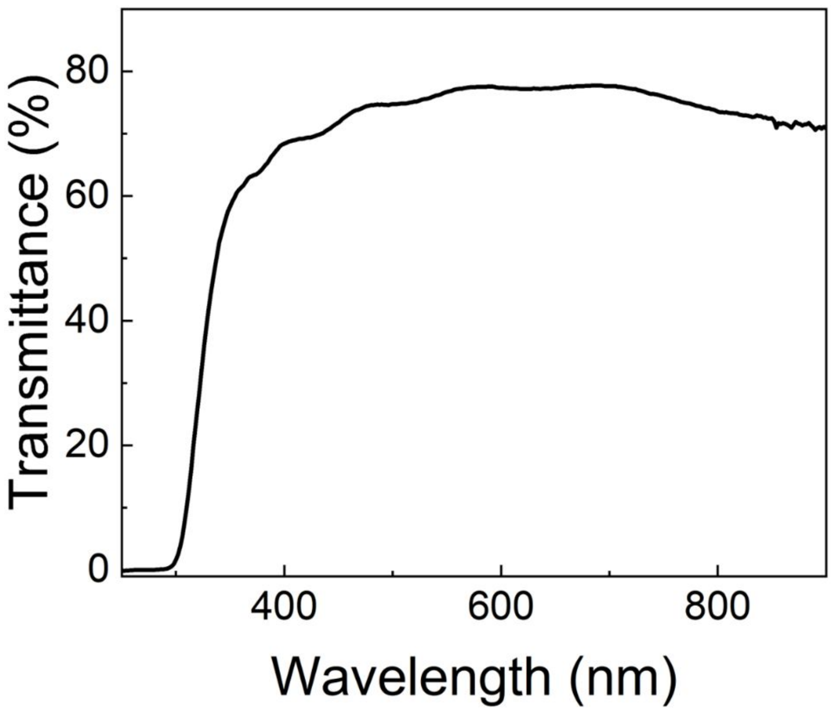

Figure S1. Transmittance spectra of glass/FTO.

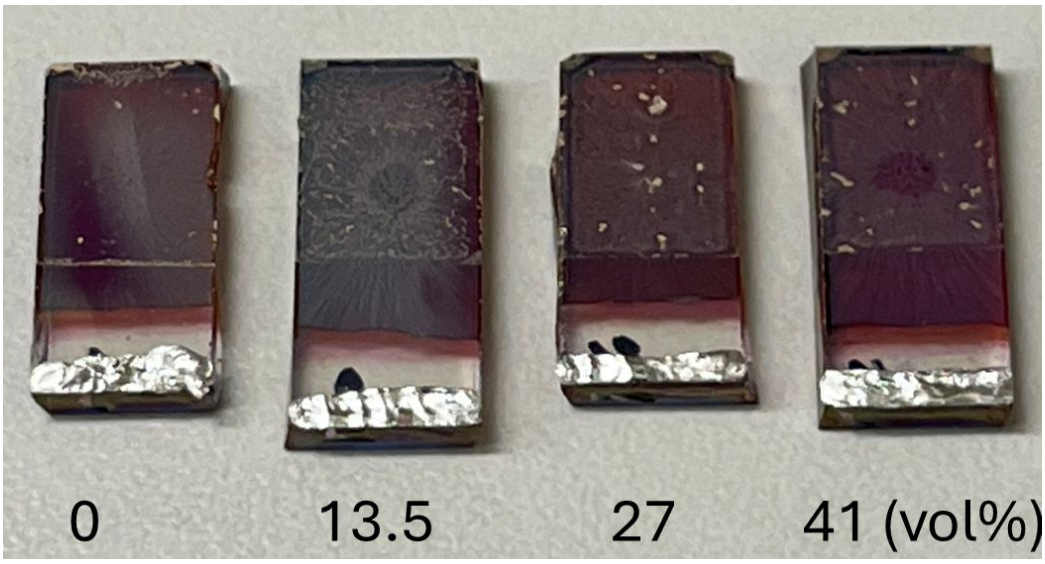

Figure S2. Digital images of glass/FTO/TiO_2/Sb_2S_3/P3HT/PAgNWs for various added P3HT concentrations in AgNWs solution.

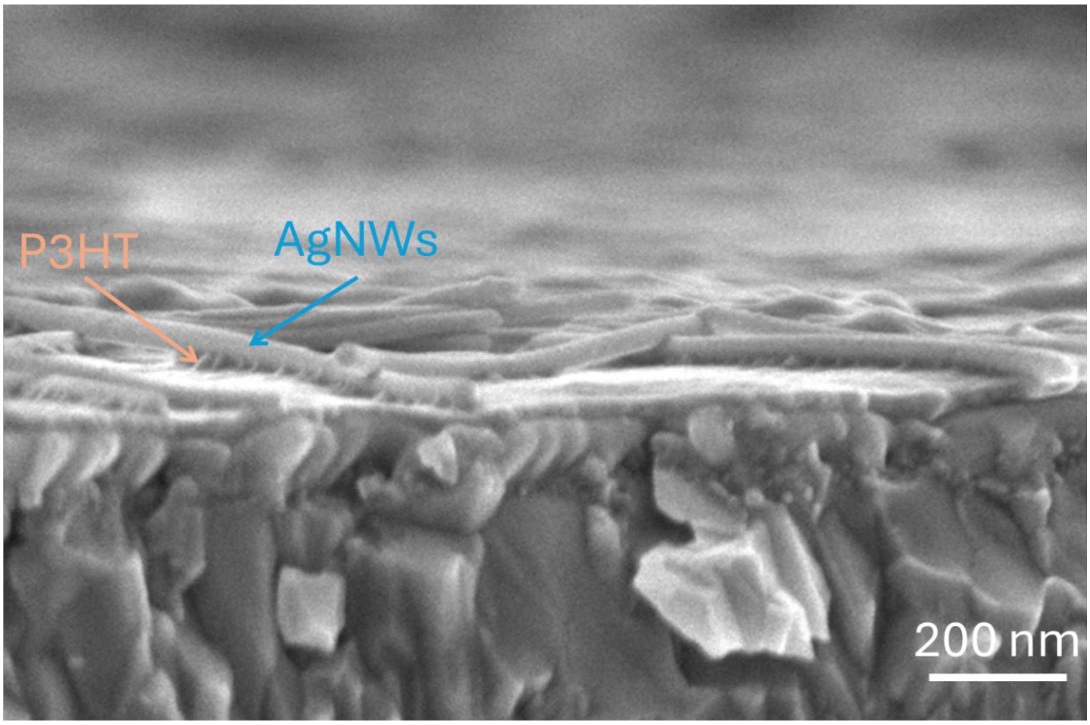

Figure S3. Tilted cross-sectional SEM image of glass/FTO/TiO_2/Sb_2S_3/P3HT/PAgNW-2.

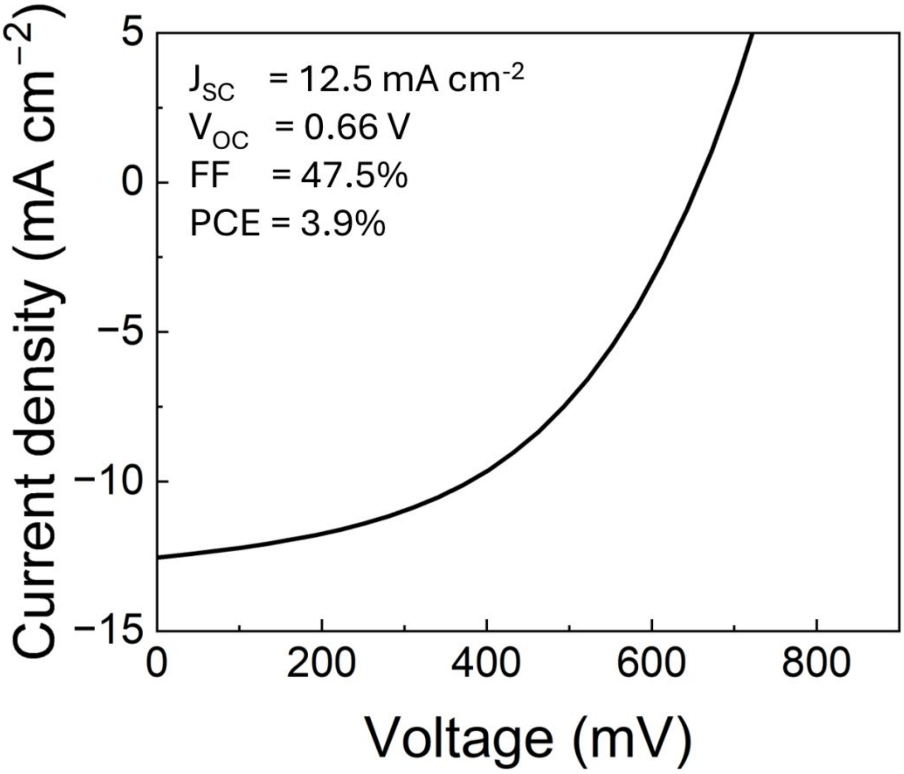

Figure S4. Current – voltage characteristics of glass/FTO/TiO_2/Sb_2S_3/P3HT/Au under simulated 1 Sun illumination.

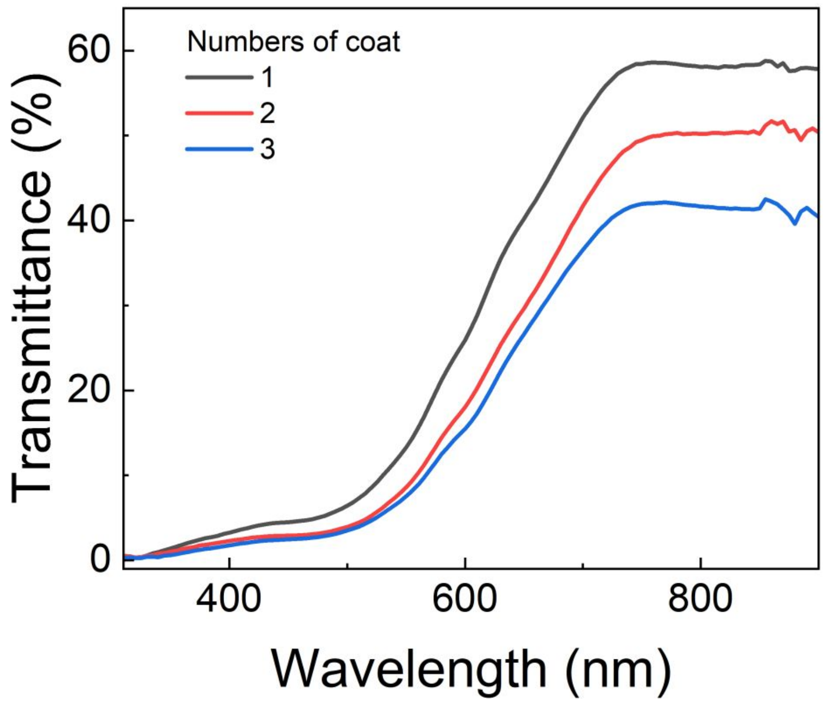

Figure S5. Transmittance profiles of glass/FTO/TiO_2/Sb_2S_3/P3HT/PAgNW-2 for various numbers of coat of PAgNW-2.

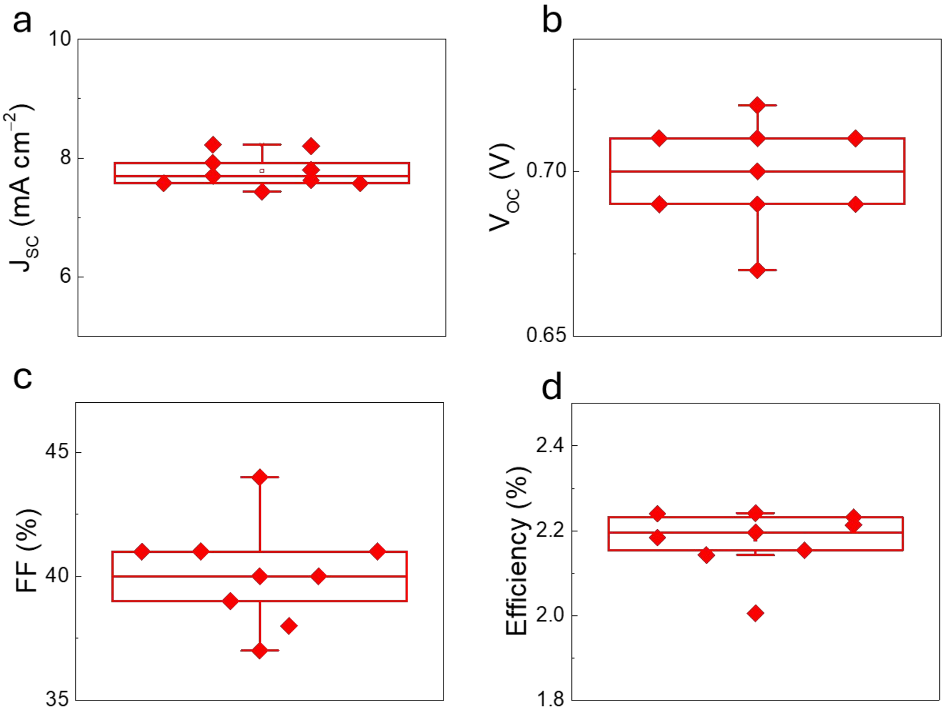

Figure S6. Statistic performance of the glass/FTO/TiO_2/Sb_2S_3/P3HT/PAgNWs device. (a) Short circuit current (J_{SC}), (b) open circuit voltage (V_{OC}), (c) fill factor (FF), and (d) efficiency.

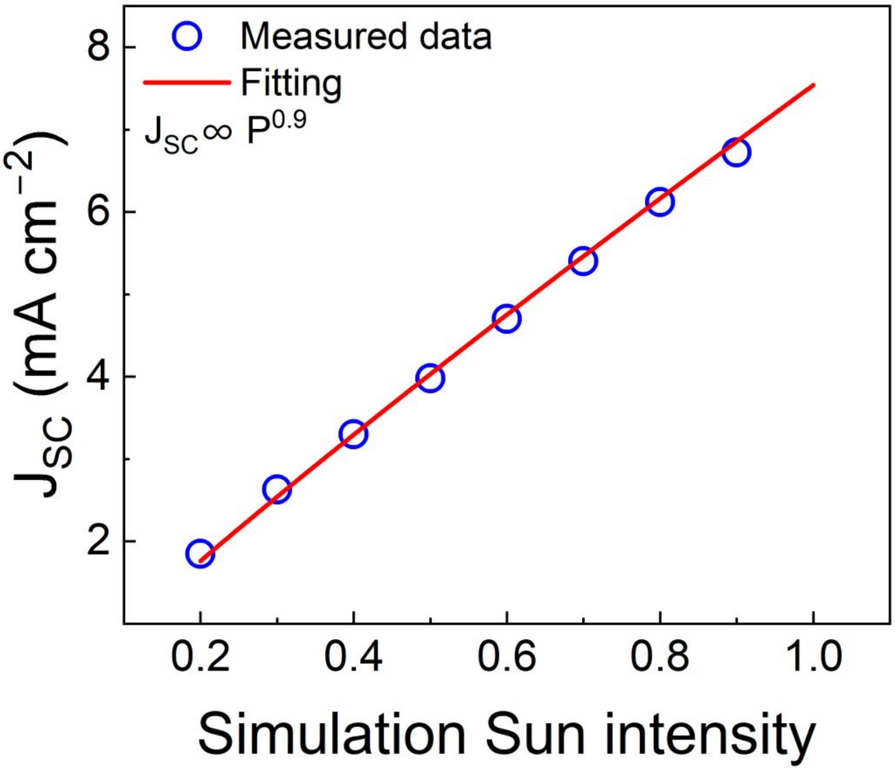

Figure S7. J_{SC} as a function of simulated Sun intensities

Video S1. An operation of a micro-motor powered by Sb_2S_3-based STPV under 1 Sun illumination.
